# Recent advances in understanding osteosarcoma and emerging therapies

**DOI:** 10.12703/r/9-18

**Published:** 2020-11-26

**Authors:** Nathalie Gaspar, Maria Eugenia Marques da Costa, Olivia Fromigue, Robin Droit, Pablo Berlanga, Antonin Marchais

**Affiliations:** 1 Department of Oncology for Child and adolescent, Gustave Roussy cancer campus. France; 2 National Institute for Health and Medical Research (INSERM) U1015, Gustave Roussy, France; 3 INSERM, UMR981, France; 4 Université Paris Sud, Orsay, France

**Keywords:** Osteosarcoma, Therapy, Precision medicine, Omics

## Abstract

Osteosarcoma is the most common bone cancer in adolescents and young adults, but it is a rare cancer with no improvement in patient survival in the last four decades. The main problem of this bone tumor is its evolution toward lung metastatic disease, despite the current treatment strategy (chemotherapy and surgery). To further improve survival, there is a strong need for new therapies that control osteosarcoma cells with metastatic potential and their favoring tumor microenvironment (ME) from the diagnosis. However, the complexity and heterogeneity of those tumor cell genomic/epigenetic and biology, the diversity of tumor ME where it develops, the sparsity of appropriate preclinical models, and the heterogeneity of therapeutic trials have rendered the task difficult. No tumor- or ME-targeted drugs are routinely available in front-line treatment. This article presents up-to-date information from preclinical and clinical studies that were recently published or presented in recent meetings which we hope might help change the osteosarcoma treatment landscape and patient survival in the near future.

## Introduction

Osteosarcoma is the most common bone cancer in adolescents and young adults (80% of the patients are younger than 25 years old) but is a rare cancer (estimated incidence of 0.2 to 3 new cases/million per year in Europe)^1^. Survival of these patients has not improved in the last four decades. The main problem of this bone tumor is its evolution toward lung metastatic disease. Patient survival decreases when lung metastasis are present at diagnosis regardless of the chemotherapy regimen used^2–5^ or when lung metastasis appeared during the disease evolution (3-year progression-free survival [PFS] is around 20%)^6–8^.

Primary tumor surgery is an important part of the treatment, as unresectable osteosarcoma has poorer survival. Osteosarcoma chemosensitivity was demonstrated in the 1970s to 1980s with response rates of 19 to 40% to methotrexate, cis-platinum, adriamycin, ifosfamide, and etoposide^9^. Since then, first-line standard treatment has not been modified and includes neoadjuvant and post-operative multi-drug chemotherapy associated with surgical resection of the primary tumor and all remaining metastatic localizations if present^10^. In addition to the presence of initial metastases, histological response of the primary tumor to neoadjuvant chemotherapy is a strong prognostic factor of relapse^4^. Intensification of chemotherapy at diagnosis or relapse has not modified patient outcome^3,11^. Extensive efforts to identify more effective or less toxic regimens, both at diagnosis and relapse, have been disappointing up to now^6^.

This emphasizes the strong unmet need for new therapies to improve survival for these patients^6^. The main objective would be to control osteosarcoma cells with metastatic potential and their favoring tumor microenvironment (ME) from the diagnosis. However, the complexity and heterogeneity of those tumor cell genomic/epigenetic and biology^12^ as well as the diversity of tumor ME where it develops (bone primary tumor and lung metastatic niche) have rendered the task difficult. No phase II trial in relapse osteosarcoma has yet been transposed to consensual successful first-line phase III trials^6^. No tumor- or ME-targeted drugs are routinely available in front-line treatment. This article presents up-to-date information that was published or presented in recent meetings and that we hope might help change the osteosarcoma treatment landscape and survival in the near future.

## The complexity of osteosarcoma biology: what are next-generation sequencing tools bringing? What remains to be solved?

In recent years, the scientific community has started to unscramble osteosarcoma complex genomic^13,14^ with next-generation sequencing (NGS), mainly at the DNA level (whole exome sequencing [WES] and whole genome sequencing [WGS]). They first characterized the considerable levels of phenotypic heterogeneity, aneuploidy, and the high rate of complex chromosomic aberrations (copy number alterations, kataegis, and chromothripsis) across the whole genome^12,13,15,16^ in osteosarcoma. Gene-centric studies have all converged to describe TP53 deficiency as the major early oncogenic event probably underestimated until the discovery of intronic TP53 mutations^17^. Few other recurrent mutations have been observed in DNA repair and cell cycle genes at the somatic (for example, RB1, ATRX, and PTEN/PI3K)^15^ and constitutional (osteosarcoma predisposing syndromes include alterations of TP53, RB1, and RECQL4)^18^ level. DNA repair and cell cycle pathways are also recurrently altered at a genomic level (for example, recurrent deletions TP53, RB1, CDKN2A/B genes, or recurrent amplifications, COPS3, CCNE1, MDM2/CDK4, MYC genes, and 6p12.3 amplifications) in addition to several receptor tyrosine kinases and downstream pathways (for example, recurrent amplifications in WNT, insulin-like growth factor 1 receptor [IGF1R], PI3K, and MAPK pathways)^14–16^. Besides genetic alterations, accumulating evidence highlights the important role of epigenetic modulation in osteosarcoma oncogenesis^12,14,19^, although this role is not fully understood.

In parallel to the emergence of new questions, the advances in osteosarcoma molecular description, achieved with NGS technology, also participated to the complexity of the general picture such as some previous unsolved problems might seem even more obfuscated now. We still have to identify the initial osteosarcoma oncogenic events with, as a subsidiary enigma, the exceptional resilience of osteosarcoma cell population to survive and expand while having such a chaotic genome. Complexity which also estranged the definition, trivial in other pathologies, of key oncogenic drivers paving disease emergence or progression to metastasis, delaying the implementation of routinely usable prognostic biomarkers or robust molecular/biological stratification able to drive future therapeutic development.

Other investigations such as transcriptomic analysis—RNA expression level and RNA sequencing (RNA-seq)—could be a better/upper read-out of what is occurring in tumor cells, tumor ME, and their interplay^20^. Indeed, the osteosarcoma transcriptomic landscape and its diversity might better sum up, as the first phenotype, the multiple chromosomal rearrangements, the epigenetic events^13,15^, but also the various tumor ME (bone primary tumor, circulating cells, and lung metastatic site) and their heterogeneity (for example, osteoforming and osteolytic areas in bone primary but also lung metastasis^21^ and hypoxic area). The interplay between tumor cells and ME cells and the identity of cells that influence osteosarcoma fate remain unclear.

To fully reveal the global osteosarcoma molecular picture, including its ME, large-scale approaches should couple integrative analyses at both the DNA/RNA level and upper levels (for example, the proteome level). Owing to the rarity of these patient samples, the collection of the optimal dataset requires an effort to merge existing genomic/biological datasets with optimal clinical annotation to ease researchers’ work in the field. Very few osteosarcoma genomic datasets are available worldwide, usually with no detailed and sparse clinical annotation, and issued from samples mainly at diagnosis (for example, the TARGET dataset and others^20,22,23^). Relapse samples, which might give access to clonal evolution and metastatic phenotype, are even rarer, but the situation is improving. Several molecular profiling programs have been set up at relapse^24,25^, and data should be available soon for the research community.

Multi-regional WES/WGS in primary and metastatic matched samples from patients with osteosarcoma has started to reveal the dynamic evolutionary process and temporo-spatial tumor heterogeneity of osteosarcoma lung metastases^26^. Thus, metastases exhibit a higher mutational burden, genomic instability, improved immunogenicity, and a more significant inter-tumoral rather than intra-tumoral heterogeneity^26^. The pro-metastatic role of tumor-associated exosomes, micro-RNA, long non-coding RNA, circular RNA, and metabolism is just starting to be explored^27^. The combination of identification and quantification of somatic alterations in plasma-derived ctDNA (circulating tumor DNA) is gaining traction as a non-invasive and cost-effective method of disease monitoring in patients with osteosarcoma, particularly to evaluate the response to treatment and monitor for disease recurrence^28^. However, prospective validations in larger cohorts are needed.

Understanding the clonal dynamics during osteosarcoma disease evolution from primary tumor to metastatic sites might be a clue for future therapeutics. Several new technologies are starting to create wider opportunities to achieve this goal. The next revolution will come from NGS moving from bulk to single-cell tumor analysis. Single-cell NGS is clearly the next step that might help us understand the osteosarcoma cell of origin, initial oncogenic event(s), and the clonal and ME dynamics along the disease progression from primary tumor, through circulating cells, toward the lung metastatic site. A near single-cell tracking system in a *de novo*–induced murine osteosarcoma model suggests that osteosarcomagenesis could follow a neutral evolution model, in which different cancer clones coexist and propagate simultaneously^29^. In the near future, single-cell whole-genome and transcriptomic analyses offer great promise in deciphering the local and global interactions driving the tumor–host dynamic within the primary tumor and the metastatic niche^30^. However, the bone ME might be challenging for single-cell isolation in human disease. In addition, single-cell resolution comes at the cost of losing the spatial organization of the tumor ME. Other technologies such as spatial transcriptomic might provide excellent integration of nearly single-cell transcriptomic with histopathological analysis. Such technology will produce detailed maps of the tumor and ME cells in each tumor sample, unravelling spatial arrangement and which types of crosstalk may occur between tumor and ME cells according to the context (bone/lung localization). This should allow a deeper understanding of the important targets in osteosarcoma.

## Targeting the tumor cells: moving toward a personalized medicine?

Although the decoding of the osteosarcoma genome greatly advanced the understanding of the genomic osteosarcoma landscape from early oncogenesis to metastatic spread properties, immediately actionable therapeutic targets are not yet obvious. So far, preclinical data have not translated to a successful targeted therapy phase II relapse trial^6^.

Up to now, only few targeted agents have been tested in a phase II trial for refractory/recurrent osteosarcomas, the setup of such trials started late compared with that of other cancer trials (>2007), and their efficacy results were disappointing^6^. The biological rationale leading to these phase II trials was usually light. When described, it relied mainly on the general mechanism of drugs/combination activity in cancers rather than on specific osteosarcoma targets (for example, mammalian target of rapamycin [mTOR] inhibitors) or on the relative expression level of osteosarcoma cell surface protein with little knowledge about its mechanistic role (for example, anti-GD2, anti-HER2). This clearly reflects the lack of strong preclinical studies in osteosarcoma at that time. More specific therapeutic development based on osteosarcoma biology is being attempted. For example, the BRCAness phenotype of osteosarcoma^22^ is being exploited as a therapeutic target for poly ADP ribose polymerase (PARP) inhibition^31^. *In vitro* results seem less impressive than what was observed in *BRCA*-deficient breast cancer^32^. Clinical relevance in patients is being evaluated in ongoing trials (for example, e-smart trial PARP inhibitor arm, NCT02813135). In addition to needing better knowledge of osteosarcoma biology, we need appropriate and available preclinical models to properly perform preclinical drug evaluation and to define the level of preclinical evidence required to study a new drug in patients. Efforts have been made in the last few years to improve preclinical testing of drugs by developing patient-derived xenograft (PDX) models from primary tumor at diagnosis or metastatic relapse, including in the bone orthotopic setting (ITCC-P4, www.itccp4.eu; NCI PPTC, www.ncipptc.org), but no minimum preclinical requirement to bring a drug to the clinic has been defined for osteosarcoma.

Targeting the initial oncogenic event or those responsible for the metastatic phenotype (or both) would be a great help in osteosarcoma. The mechanism facilitator of lung metastasis remains to be identified from tumor cells, primary bone tumor ME, or lung metastatic ME; they may represent clinically relevant targets. Preclinical proof of concept of personalized medicine targeting osteosarcoma abnormalities was shown in PDX models. Orthotopic osseous osteosarcoma PDX models issued from primary tumor at diagnosis or metastatic relapse retain the tumor characteristics of the patient sample they are issued of, with the best clonal preservation across different pediatric tumors, as well as cellular features of the patient tumor and the epigenetic landscape of their developmental origins^33^. Via such PDX models and an integrated approach (WGS and matched RNA-seq) to identify somatic copy number alterations with the most highly amplified genes, a limited set of copy number patterns can group osteosarcoma tumors into subtypes that may predict response to certain targeted agents (for example, genome-informed targeting of MYC/CDK9, Cyclin E/CDK2, CDK4/6, PI3K/AKT/PTEN/mTOR, AURKB, and VEGF pathways)^34^. These data can be used to inform new agent prioritization decisions for drug development in osteosarcoma and open the field of cell cycle inhibitors as anti-osteosarcoma drugs along with other publications. However, these altered patterns explain only a small number of events, and the degree of cell-to-cell heterogeneity within a tumor remains unknown. Furthermore, translation to humans remains to be proven. Several molecular-driven therapeutic programs are ongoing (for example, e-SMART, NCT02813135; INFORM2, NCT03838042; and the MATCH Screening Trial, NCT03155620), including with drugs of potential interest in osteosarcoma (e-SMART, NCT02813135, CDK4/6 inhibitor arms), and results are eagerly awaited. These programs are based mostly on WES abnormalities while gene expression by RNA-seq is usually not taken into account. This might be thought out differently in the future.

## Targeting the microenvironment: hope or despair? Moving toward a combined or an adapted ME strategy?

The genomic complexity and tumor heterogeneity of osteosarcoma as well as the importance of ME (osseous, angiogenic, and immune ME) in osteosarcoma metastatic phenotype and outcome have orientated drug development toward ME-directed drugs at a different level for the last two decades. Some efficacy of these strategies has been observed in phase II trials at relapse. Fewer attempts to introduce such drugs then in first-line treatment have not yet been successful, but some are still ongoing. Again, the reliability of the preclinical models that do not recapitulate the whole human ME is questionable. Humanized models or syngenic models, including spontaneous osteosarcoma in dogs, are being explored.

### Osteosarcoma immune microenvironment

Osteosarcoma is a macrophage-dependent tumor with few lymphocytes present in its hostile hypoxic tumor ME. Tumor immunity plays an important role in osteosarcoma metastatic behavior. Localized tumors at diagnosis present high tumor-infiltrating macrophages (TAMs) of M1 polarisation associated with a low rate of tumor-infiltrating lymphocytes (TILs) and a balance in favor of CD8 effectors^35,36^. In contrast, primary tumors issued from metastatic patients present M2-polarised TAMs with immunosuppressive, tissue remodeling, and pro-angiogenic properties^37^ and also exhausted/anergic CD8^+^ TILs^38^ and a balance favoring the immunosuppressive FOXP3^+^ T regulator (Treg)^36^. This pro-tumor immune contexture appears to be enhanced in lung metastasis samples^35,39^. Rather than a clear dichotomic situation, a continuum between both states due to ME heterogeneity might better mimic the reality of osteosarcoma immune ME, explaining some apparent conflicting results^40^. The relative role of the tumor immune profile or the patient immune profile is not yet understood. Owing to this osteosarcoma immune ME, immune therapy has been considered an excellent choice for targeting osteosarcoma metastatic phenotype.

Therefore, targeting the intra-tumor macrophage environment by liposomal mifamurtide (L-MTP-PE, MEPACT^®^) as an immune modulator able to activate monocytes/macrophages was promising in phase II^41^. The controversial results on mifamurtide efficacy associated with post-operative chemotherapy, issued from the Intergroup INT-0133 phase III study, in localized osteosarcoma^42,43^ and the insufficient power of the analysis performed separately in metastatic patients^44^, have not led to a homogenized international use of this drug. The US Food and Drug Administration did not approve the drug, whereas the European Medicines Agency approved the drug for localized osteosarcoma, but mifamurtide is still not reimbursed in all European countries. The current first-line French sarcoma 13/OS2016 (NCT03643133) randomized phase II trial in high-risk osteosarcoma (distant/skip metastatic disease at diagnosis and localized disease with poor histological response) might help to resolve this question^45^. Interferon alfa failed to show efficacy and was considered toxic in a first-line phase III EURAMOS trial^2^. Lymphocyte-targeted immune therapies with check-point inhibitors (anti-PD1/PDL1) were disappointing in refractory/relapsed osteosarcoma phase I/II trials with the majority of the patients experiencing progressive disease when anti-PD1/PDL1 was given either as single agent^46,47^ or combined with metronomic cyclophosphamide^48^. However, recent data comparing the metastatic and primary tumor niches^30^ suggested that relapse disease might not be the best population to test such drugs, and anti-PDL1 is now being tested as maintenance after adjuvant first-line chemotherapy (NCT03676985).

### Osteosarcoma vascular microenvironment

Angiogenic pathways—for example, vascular endothelial growth factor receptor (VEGFR) and platelet-derived growth factor (PDGF)—have been implicated in osteosarcoma tumor evolution and linked with their metastatic behavior and poor prognosis. Targeting the tumor vascular environment with multi-tyrosine kinase inhibitor harboring anti-angiogenic activity (targeting VEGFR, PDGF receptor, and fibroblast growth factor receptor) is the most promising therapeutic option at the moment in relapsed/refractory osteosarcoma but with no known biomarker of efficacy. Whether the anti-osteosarcoma activity is due only to their angiogenic properties or to an additional role on tumor cells is still unknown. Several multi-tyrosine kinase inhibitors have been tested as a single agent in both adult/pediatric populations (regorafenib randomized phase II against placebo^49,50^; single-arm phase II trials of sorafenib^51^, apatinib^52^, cabozantinib^53^, and lenvatinib^54^) with a class effect efficacy (median PFS ranged between 3 and 6 months, whereas median PFS for placebo was 4 weeks). The main side effects (for example, hypothyroidy, hypertension, and proteinuria) are usually manageable by dose reduction and symptomatic measures. The relation between efficacy and observed side effects is not clear. No unanimous biomarker of efficacy exists. Pneumothorax is observed in 6 to 17% of patients with lung metastases. This seems to be a class effect, specific to osteosarcoma, as it is extremely infrequent in other tumor types (incidence <1%). Currently, the regorafenib single agent is being introduced in first-line treatment as maintenance treatment after the end of the conventional chemotherapy for patients in complete remission (REGOSTA, NCT04055220). Unfortunately, although data on efficacy/toxicity in children/adolescents are available, the REGOSTA trial includes only patients who are 16 years old and above. Combining this drug with others might be challenging. The combination sorafenib/mTOR inhibitor was poorly tolerated and did not exhibit increased efficacy^55^. The combination lenvatinib+VP16/ifosfamide was feasible with acceptable toxicity, mainly chemotherapy-related^54^. A randomized phase II trial VP16/ifosfamide with or without lenvatinib in relapsed osteosarcoma has just opened (OLIE, NCT04154189).

### Osteosarcoma bone microenvironment

Osteosarcomas are characterized by the direct formation of osteoid matrix by tumor cells^56^, associated with osteolytic lesions. The invasion of bone tissue by tumor cells, through their ability to deregulate bone remodeling, affects the balance between bone resorption (osteoclasts) and bone formation (osteoblasts). A vicious cycle between tumor and bone cells is described during osteosarcoma development. Cancer cells produce soluble factors, such as cytokines—for example, interleukin-6 (IL-6), IL-11, tumor necrosis factor alpha (TNF-α), and receptor activator of nuclear factor kappa-Β ligand (RANKL)—that activate osteoclastogenesis, leading to bone degradation. Following bone resorption, growth factors trapped in the bone matrix, such as IGF-1 or transforming growth factor beta (TGF-β), are released in the bone ME and stimulate tumor growth^57^. Targeting the osteoclastic activity was thought to be an interesting target in osteosarcoma.

Despite preclinical marked evidence in mouse/rat models^58,59^, zoledronate, a biphosphonate with anti-osteoclastic activity, failed to show added efficacy to chemotherapy in the French first-line OS2006 phase III trial^60^, confirmed by a Chinese trial^61^. One hypothesis for the lack of efficacy in patients with osteosarcoma is a potential deleterious effect of zoledronate on the immune system^40^ and/or the heterogeneity of tumor ME, not properly captured in animal models^21^. In a phase II trial (NCT02470091) in the US, the RANKL inhibitor denosumab (Prolia^®^) also had insufficient activity in refractory/recurrent osteosarcoma for further development^62^.

### Osteosarcoma lung microenvironment

The bone ME seems to be reproduced in osteosarcoma lung metastatic foci, and differences compared with primary tumor are observed, especially in the immune ME, which seems more immunosuppressive in metastasis^30,35^. In addition, very little is known about the biology that drives lung colonization and the specificity of the lung niche leading to osteosarcoma proliferation or dormancy^63^. More should be understood to use it as a therapeutic target.

### How to move forward with osteosarcoma tumor microenvironment

In the future, simultaneously targeting several aspects of the osteosarcoma ME might be more efficient than targeting only one aspect. Preclinical evidence suggests increased anti-osteosarcoma efficacy when zoledronate and L-MTP-PE are associated^64^. A French clinical phase II trial in sarcoma including osteosarcoma, combining both an anti-PD1 and a multi-tyrosine kinase inhibitor with angiogenic activity, should open soon. Another option would be to tailor ME modulation therapies to the ME heterogeneity of each individual tumor. This last strategy will require reliable markers of the tumor ME characteristics and is a field of further development.

## Drug development in osteosarcoma: how to improve trial design

In this review, we have discussed several key elements to speed up drug development in osteosarcoma: a better biological understanding of tumor cells, tumor ME, and metastatic processes; more reliable preclinical models reflecting tumor cell heterogeneity and tumor ME; and the need to identify prerequisites to bring a drug for preclinical testing to patients.

Another important question is how to optimize trial design to rapidly evaluate the efficacy of a given drug/combination and to improve patient survival.

Up to now, phase II trials have been developed in refractory/relapsed osteosarcoma, with an inconsistent go or no-go decision to bring the drug in a first-line phase III trial, as no historical data were reliable. The increased number of new targeted and immune therapies have led to an increased number of trials testing these drugs, while the place of standard chemotherapy in relapse treatment is not properly defined. Most of the time, the choice of drugs to be introduced in refractory/relapsed osteosarcoma phase II trials had no specific osteosarcoma rationale^6^, and when present, the rationale is based on primary tumor biology rather than metastatic biology. The increased availability in PDX models issued from relapsed metastatic disease might help to inform molecular-driven therapies, find biomarkers of efficacy, and possibly understand mechanisms underlying lack of efficacy but these immunodeficient models might not be as useful for immunotherapies. The number of models to be tested is a matter of debate. In addition, different relapse presentation might not benefit a therapy in the same way (for example, bulk disease versus minimal residual disease and lung versus bone metastasis).

Ideally, randomized phase II trials against placebo or standard treatment would help determine drug efficacy^49,50^, as the heterogeneity of the population can be taken into account by stratification. However, these randomized trials require more patients and thus are longer than single-arm trials. Bayesian trial designs might be of value in rapidly evaluating multiple treatments for rare tumors as done in Ewing sarcoma (rEECur trial)^65^ and to integrate previous historical data (sarcoma 13 trial)^45,66^, tying to minimize the number of patients required.

An analysis of negative phase II trials was suggested to be used as historical control to rapidly evaluate future clinical trials in refractory/relapse osteosarcomas^67^. Using this approach, trials could be conducted in less than 3 years^68^. However, this analysis was based on refractory/relapsed osteosarcomas with either measurable disease according to RECIST (soft tissue component lesion greater than 1 cm) or disease in complete remission after surgery. Patients with only evaluable disease were left out of this approach.

The use of biomarkers to classify individuals into smaller, biologically related groups would dramatically affect our approach to conducting clinical trials but might help to get better signal of efficacy. A basket trial based on molecular tumor characteristics across cancer types might be a useful approach to get efficacy signal.

In addition, the extrapolation of efficacy response in patients with refractory/relapsed osteosarcoma to patients with newly diagnosed disease is not established. Depending on the mechanism of the drug, the appropriate population to test a given drug might not be a population with relapsed disease. Thus, discussions on how to design trials and in which population the drug should be tested are critical for drug development in osteosarcoma, as well as preclinical evidence to bring a drug in the clinic, with more than one way of doing it.

## Closing summary

To conclude, increasing osteosarcoma survival in the future will require broad expertise from key stakeholders from biology/bio-informatics to clinic and statistics and a joint effort to collect/connect all the available information on biology and clinical efficacy and build up new innovative strategies to test new drug efficacy in patients.
